# Ceftriaxone-Induced Immune Hemolytic Anemia and Subsequent Disseminated Intravascular Coagulation

**DOI:** 10.7759/cureus.103776

**Published:** 2026-02-17

**Authors:** Hunter T Pham, Jennifer Bach, Christian Kolacki, Jeffery S Jones, Nathan DeBruine

**Affiliations:** 1 Emergency Medicine, Michigan State University College of Human Medicine, Grand Rapids, USA; 2 Emergency Medicine, Corewell Health, Grand Rapids, USA; 3 Emergency Medicine, Spectrum Health Medical Group, Grand Rapids, USA

**Keywords:** ceftriaxone, ceftriaxone adverse effects, dic, diiha, disseminated intravascular coagulation, disseminated intravascular coagulation (dic), drug-induced immune hemolytic anemia, hemolytic anemia, immune hemolytic anemia, serology

## Abstract

Drug-induced immune hemolytic anemia (DIIHA) is a rare but potentially life-threatening complication, most often associated with β-lactam antibiotics such as ceftriaxone. We report a 78-year-old woman who developed abrupt clinical deterioration and disseminated intravascular coagulation (DIC) within 30 minutes of ceftriaxone administration for pneumonia, resulting in death eight hours after exposure despite aggressive resuscitation. Laboratory studies confirmed ceftriaxone-dependent antibodies and a positive direct antiglobulin test (DAT), establishing the diagnosis of ceftriaxone-induced DIIHA with secondary DIC. This case underscores the fulminant and often fatal course of ceftriaxone-induced hemolytic anemia and highlights the critical importance of swift recognition and discontinuation of suspected offending agents in patients presenting with acute hemolysis and coagulopathy following antibiotic therapy.

## Introduction

Drug-induced immune hemolytic anemia (DIIHA) is an uncommon but serious adverse reaction to medications, most frequently linked to certain antibiotics. Its clinical presentation can range from mild hemolysis to a fulminant, life-threatening condition. The underlying mechanism involves drug-dependent antibodies (DDABs) that cause rapid destruction of red blood cells, detectable only in the presence of the offending drug, as extensively described by Dr. George Garratty [1]. Ceftriaxone, a commonly employed cephalosporin antibiotic in emergency departments for broad-spectrum coverage, is the second-most frequently implicated agent in DIIHA, after cefotetan [1]. Emergency clinicians should be vigilant for signs of acute hemolysis following antibiotic administration as prompt recognition and discontinuation of the causative agent are critical to patient outcomes. We report a case of a 78-year-old woman who developed rapid-onset ceftriaxone-induced immune hemolytic anemia complicated by disseminated intravascular coagulation (DIC), culminating in death. The diagnosis was retrospectively confirmed by detection of ceftriaxone-dependent antibodies. This case highlights the importance of early identification of DIIHA in the emergency setting to guide treatment decisions and potentially improve survival.

## Case presentation

A 78-year-old woman with multiple chronic conditions, including type 2 diabetes mellitus, presented to a rural emergency department with sudden onset of nonspecific symptoms after awakening from a nap, including drowsiness, one episode of vomiting, and generalized abdominal pain. Emergency medical services also reported a leftward lean and right facial droop, which improved with facial muscle activation.

Vital signs at the time of arrival were remarkable for a temperature of 36°C, a heart rate of 96 beats per minute, a respiratory rate of 18 per minute, a blood pressure of 151/90 mmHg, and oxygen saturation of 98% on room air. Physical examination revealed dry mucous membranes, left lower quadrant abdominal tenderness to palpation, and grossly normal lung and heart sounds on auscultation. Neurological examination was remarkable for apparent left-sided facial droop, transient bilateral upper extremity ataxia, and generalized weakness. The patient reported left-sided sensory deficits. There was no appreciable extremity drift or visual field deficit.

**Figure 1:**
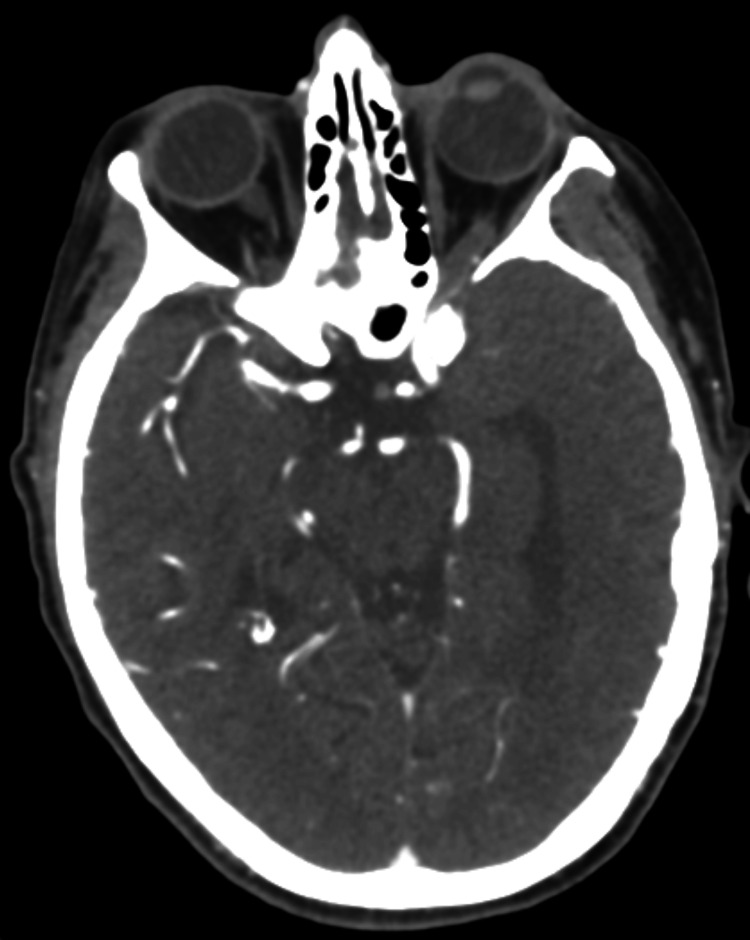
CT Angiogram of the Brain (Axial View) CT angiography of the brain is grossly unremarkable, with no overt abnormal findings. CT: computed tomography

**Figure 2:**
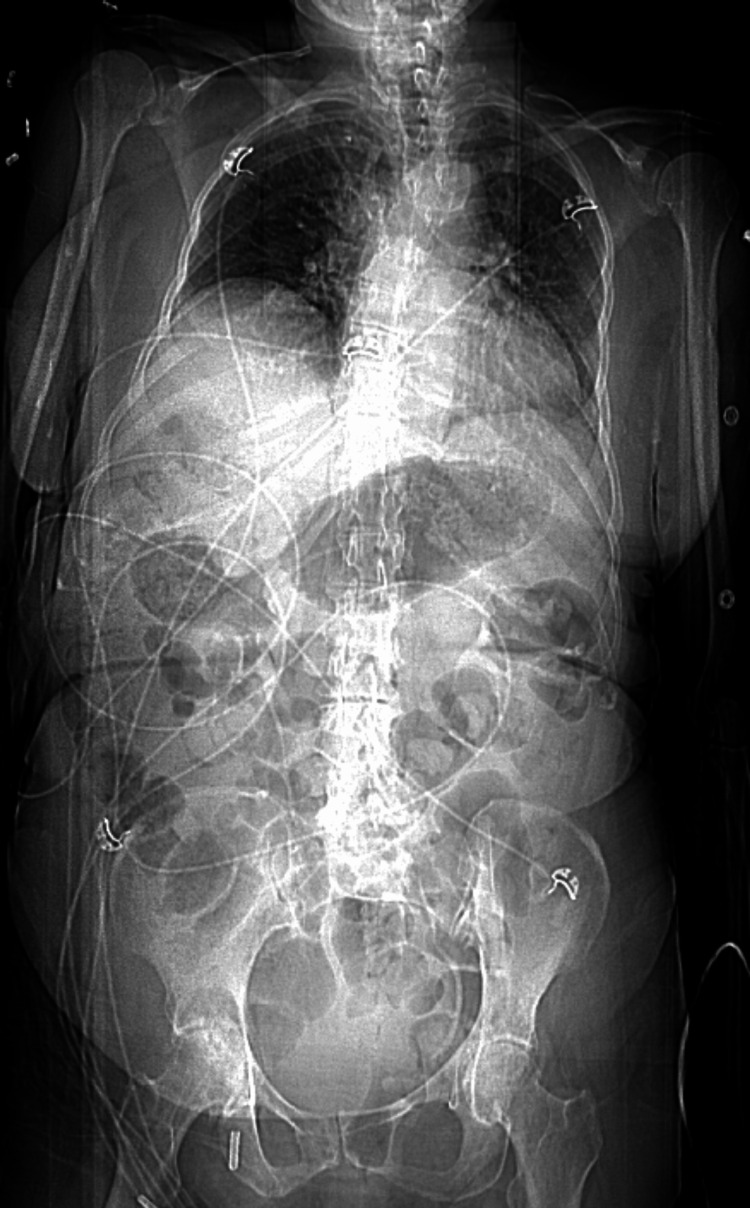
CT of the Thorax/Abdomen/Pelvis (Coronal View) Coronal view of the CT of the thorax/abdomen/pelvis does not reveal overt abnormalities. CT: computed tomography

**Figure 3:**
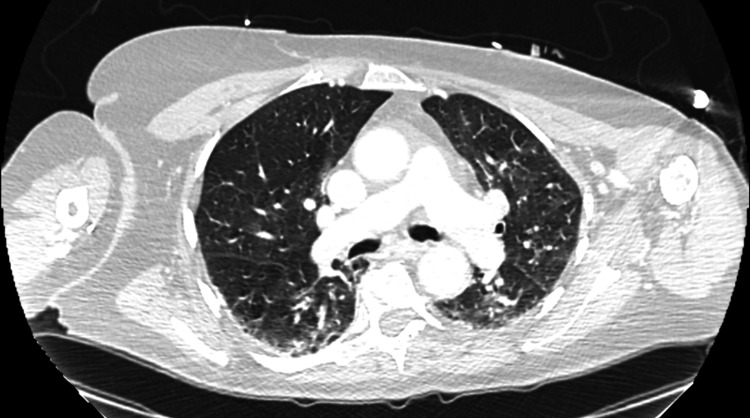
CT of the Thorax (Axial View) Axial view of the CT of the thorax/abdomen/pelvis revealed some prominence of interstitial lung markings, which is nonspecific but can be consistent with pneumonia. CT: computed tomography

A stroke code was activated with a National Institutes of Health Stroke Scale (NIHSS) score of 8 and a last-known-well time greater than five hours prior to arrival. Initial laboratory workup, noncontrast computed tomography (CT) of the head, CT angiogram of the head and neck, and CT perfusion of the brain were all grossly unremarkable (Figure 1). After an episode of emesis, further physical examination revealed left lower quadrant abdominal tenderness. Additional CT imaging was obtained, considering septic metabolic encephalopathy as an etiology of the patient’s drowsiness. CT of the thorax/abdomen/pelvis with contrast demonstrated mild prominence of interstitial lung markings suspicious for an infectious process with no other acute findings (Figure 2 and Figure 3). With those interstitial lung markings on CT imaging, empiric coverage of pneumonia was pursued with ceftriaxone and azithromycin.

**Figure 4:**
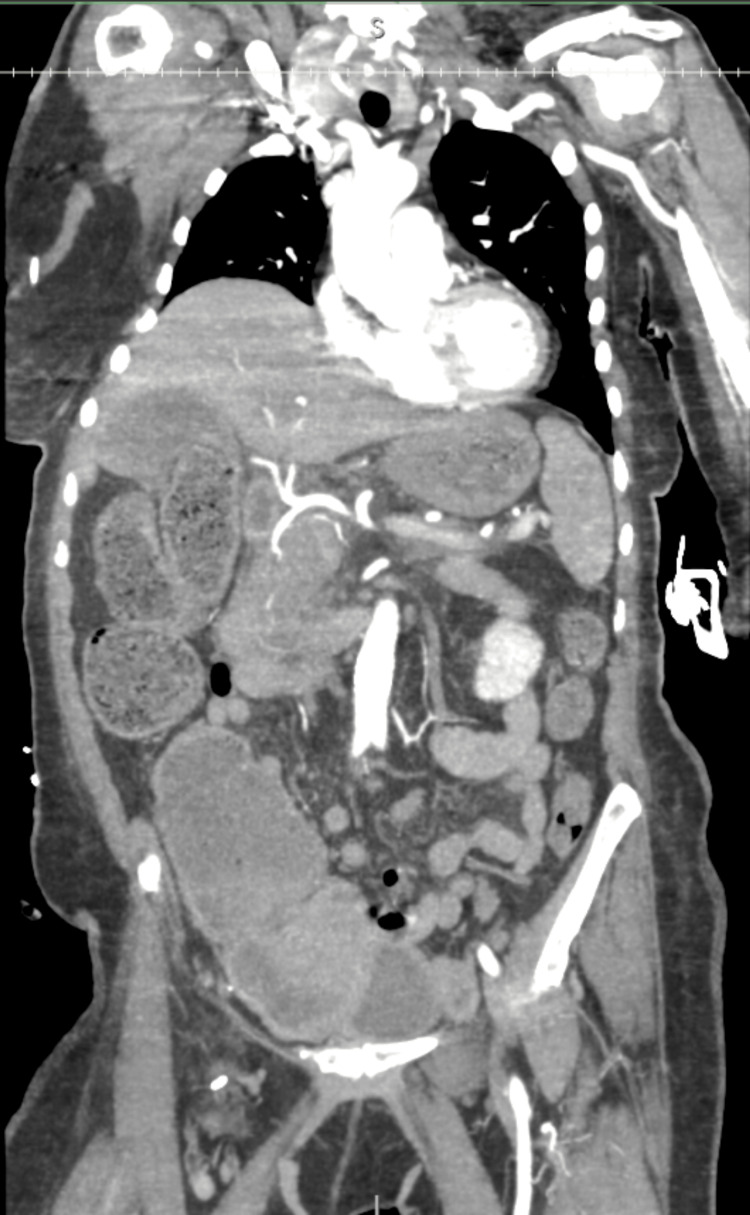
CT Angiogram of the Thorax/Abdomen/Pelvis (Coronal View) CT angiography of the thorax/abdomen/pelvis does not reveal any overt extravasation and is grossly normal. CT: computed tomography

Thirty minutes after administration of ceftriaxone, the patient experienced sudden clinical deterioration with significant hypotension at 73/52 mmHg, tachycardia at 109 beats per minute, and pallor. Point-of-care hemoglobin 33 minutes after administration of ceftriaxone revealed a drop to 7.1 g/dL from the initial 11.9 g/dL at the time of arrival three hours earlier. Femoral central venous access was established, and four units of uncrossmatched packed red blood cells were transfused, leading to an overall improvement in hemodynamics. Prior to administration of those blood products, but now after administration of ceftriaxone, a blood sample was acquired and sent for type and screen. Once the patient was stabilized, a CT angiogram of the thorax/abdomen/pelvis was obtained and revealed no overt abnormality (Figure 4). The patient was then transferred to an academic medical center 30 miles away.

Upon arrival at the academic medical center, the patient had notable lower extremity mottling. A new type and screen revealed AB+ blood type with a positive antibody screen. The initial type and screen collected prior to transfer had still not resulted, likely secondary to gross hemolysis, and was therefore not available for comparison. Oozing of blood was noted around the patient’s femoral central line, with laboratory analysis, now five hours after presentation, concerning for DIC. Repeat laboratory analysis three hours later, now six hours after administration of ceftriaxone, demonstrated worsening coagulopathy (Table 1).

**Table 1:** Laboratory Analysis Timeline

Time	Reference range	Hour 0	Hour 1	Ceftriaxone administered							
				Hour 2	Hour 3	Hour 4	Hour 5	Hour 6	Hour 7	Hour 8	Hour 9
Venous pH	7.32-7.42	7.40	-	-	7.35	7.27	-	-	7.32	-	7.04
Point-of-care bicarbonate (mmol/L)	22-32	24	-	-	22	21	-	-	19	-	18.9
Point-of-care hemoglobin (g/dL)	12-16	12.6	-	-	7.1	9.2	-	-	14.3	-	-
Hemoglobin (g/dL)	12-16	11.9	-	-	-	-	10	-	-	-	-
Platelet (×10⁹/L)	140-400	362	-	-	-	-	176	-	-	-	-
Prothrombin time (seconds)	9.5-12.0	11.3	-	-	-	-	34.1	-	-	>90	-
International normalized ratio	0.9-1.2	1	-	-	-	-	3.4	-	-	>8	-
Activated partial thromboplastin time (seconds)	24-34	21.2	-	-	-	-	58.6	-	-	67.2	-
Bicarbonate (mmol/L)	21-29	23	-	-	-	-	-	-	14	-	15
Anion gap (mmol/L)	5-14	10	-	-	-	-	-	-	18	-	22
Lactic acid (mmol/L)	<2	-	1.5	-	-	-	-	5.3	-	-	8.9
D-dimer (ng/mL)	<500	-	-	-	-	-	>35,000	-	-	-	-
Fibrin degradation products (mcg/mL)	<5	-	-	-	-	-	>20	-	-	-	-
Lactate dehydrogenase (U/L)	100-220	-	-	-	-	-	-	-	-	-	>1,800

With diffuse skin mottling and oozing from intravenous access sites raising clinical concern for DIC, empiric piperacillin-tazobactam and vancomycin were administered, and two units of fresh frozen plasma were transfused. Vital signs remained critical with a blood pressure of 86/66 mmHg, heart rate of 114 beats per minute, and development of a fever at 38.3°C. Another repeat blood draw was attempted, but results were delayed due to the blood forming “gel” in the collection tubes. The patient suffered cardiac arrest shortly thereafter, with pulseless electrical activity. Cardiopulmonary resuscitation was attempted for 25 minutes, at which time blood work prior to the arrest revealed a lactic acid of 8.9 mmol/L and a pH of 7.04, prompting termination of resuscitative measures and transition to comfort care with family present at bedside. The patient was declared deceased seven minutes later. Blood draw during resuscitation subsequently demonstrated a lactate dehydrogenase level of >1,800 U/L.

Retrospective type and screen of the patient’s initial blood draw on arrival to the community hospital demonstrated AB+ blood type and a negative antibody screen. The positive antibody screen following administration of ceftriaxone during this encounter, as well as rapid hemolysis and escalating coagulopathy, supports a diagnosis of DIIHA with secondary DIC. This rare but catastrophic reaction contributed directly to the patient’s rapid deterioration and death, although her initial presentation was of an unclear cause.

Of note, this patient had a history of recurrent prosthetic knee infections, treated with piperacillin-tazobactam, ampicillin-sulbactam, azithromycin, and metronidazole. She also had numerous hospitalizations for urinary tract infections, generally treated with ceftriaxone. During a hospitalization just three months prior to this case, she received ceftriaxone with no reported complications.

## Discussion

The antibody responsible for DIIHA is typically categorized into drug-dependent antibody (DDAB) or drug-independent antibody (DIAB). The case described here is one of drug dependence, with antibodies dependent on ceftriaxone. DDAB-related hemolysis is best evidenced by a positive direct antiglobulin test (DAT) [2], most sensitive for not only anti-C3d but also anti-IgG and rarely anti-IgA [3,4]. In this case, our laboratory did detect both anti-C3d and anti-IgG on retrospective DAT. A definitive diagnosis of DIIHA can be provided through identification of the DDAB itself, which was possible through our immunohematology laboratory. As described in this case, ceftriaxone-induced antibody was detected in the blood samples obtained after administration of ceftriaxone, but retrospective analysis of a historic blood sample obtained before administration demonstrated a negative antibody screen. The positive anti-C3d and anti-IgG on retrospective DAT provides strong evidence for DIIHA. The negative antibody screen before administration of ceftriaxone, with identification of ceftriaxone-induced antibody after administration of ceftriaxone, provides evidence of ceftriaxone-mediated DIIHA. Coagulation studies, fibrin degradation products, fibrinogen activity levels, D-dimer, and lactate dehydrogenase provide strong evidence for DIC following DIIHA. Unfortunately, the patient did not survive long enough to establish this diagnosis, but it is unlikely that the diagnosis would have changed the clinical course. As ceftriaxone-induced antibodies were not detectable before administration of ceftriaxone, it was not predictable that the patient would develop DIIHA, especially considering her tolerance of ceftriaxone multiple times in the past. The treatment for DIIHA is to stop the offending agent [5], which was only administered the one time in this case, and to consider steroids, intravenous immunoglobulin, and plasma exchange in case of warm autoimmune hemolytic anemia (wAIHA) [2]. Detection of ceftriaxone-mediated antibodies makes wAIHA unlikely in this patient, and there is limited evidence to support these additional steps in DIIHA.

DIIHA is unpredictable in both its onset and timeline. In this case, the patient notably had previous exposures to ceftriaxone without reaction, most recently during a hospitalization three months prior to this case. Demonstrating an unpredictable timeline after the onset of DIIHA, this patient deteriorated over the course of 30 minutes and was declared deceased only eight hours after administration of ceftriaxone. There are documented cases of DIIHA taking five days to manifest [6,7] or even 11 days to manifest [8]. Those cases that took longer to manifest were typically milder, noting only post-infusion chest pain, nausea, and sweating [9]. Although it is not universally fatal, it is a devastating disease in its acute form, which can be fatal in greater than 20% of cases [4] and develop into transitory hepatic or renal failure in others [4,7,10]. For survivors of DIIHA, it may be beneficial to conduct thorough cross-reactivity serological studies to ensure no future DIIHA is instigated by structurally similar cephalosporins [11].

DIIHA remains exceptionally challenging to recognize in real time, particularly in the emergency department, where its early manifestations may be obscured by underlying infection, sepsis, or complex comorbidities. This case underscores the importance of maintaining a high index of suspicion for DIIHA in any patient who develops abrupt, unexplained hemolysis or intravascular coagulopathy shortly after antibiotic exposure, even when the patient has tolerated the drug previously without incident. Serial testing and prompt communication with the blood bank are critical, as the transition from a negative pre-exposure antibody screen to a positive post-exposure result strongly supports a drug-dependent mechanism. In the acute setting, clinicians should consider empiric cessation of the suspected agent before confirmatory testing is available. Although discontinuation of the offending drug remains the cornerstone of management, additional supportive therapies, such as transfusion, plasma exchange, or immunosuppressive strategies, should be tailored to individual severity and clinical trajectory. Clinician education about the rare but severe complications of commonly used antibiotics, coupled with robust adverse event reporting, is essential to improving recognition and prevention. Ultimately, multicenter surveillance efforts and registry development are needed to better define risk factors, timelines, and effective interventions for DIIHA in emergency care. Especially in light of its documented high morbidity and mortality, incorporating DIIHA considerations into emergency department protocols for acute hemolytic anemia may improve outcomes.

## Conclusions

This case highlights a rare but devastating presentation of ceftriaxone-mediated DIIHA, which rapidly progressed to DIC and resulted in the patient’s death only eight hours after drug administration. This is strengthened by laboratory evidence of drug involvement with retrospective DAT analysis and evidence of hemolytic anemia with a rapid drop in hemoglobin and remarkably high levels of D-dimer and lactate dehydrogenase. Although ceftriaxone is commonly used and generally well-tolerated, this report underscores the potential for abrupt, unpredictable, and fatal immune-mediated reactions even in patients with prior tolerance. The diagnosis of DIIHA was retrospectively confirmed by the presence of ceftriaxone-dependent antibodies, along with supportive serological findings and hemolytic markers. The rapidity of clinical decline in this case, beginning within 30 minutes of ceftriaxone administration, emphasizes the unpredictable nature of DIIHA and the importance of early consideration in a patient with acute hemolysis following drug exposure. While there are no widely available pre-administration screening methods for drug-dependent antibodies, awareness of this condition is crucial for early recognition to facilitate rapid intervention. Further research is needed to develop diagnostic tools and management strategies for this rare but life-threatening syndrome.
